# Microarray profile of circular RNAs identifies hsa_circ_0014130 as a new circular RNA biomarker in non-small cell lung cancer

**DOI:** 10.1038/s41598-018-21300-5

**Published:** 2018-02-13

**Authors:** Shaoyan Zhang, Xiaoli Zeng, Ting Ding, Lin Guo, Yulong Li, Songlei Ou, Hui Yuan

**Affiliations:** 1 Department of Thoracic Surgery, Beijing Anzhen Hospital, Capital Medical University, Beijing, 100029 China; 2 Department of Clinical Laboratory, Beijing Anzhen Hospital, Capital Medical University, Beijing, 100029 China

## Abstract

Accumulating evidence has revealed that aberrant Circular RNAs (circRNAs) expression plays important roles in carcinogenesis and tumor progression. However, their role in non-small cell lung cancer (NSCLC) remains unclear. In this study, we first used circRNA microarrays to screen for tumour-specific circRNA candidates in between NSCLC (n = 3) and adjacent lung (n = 3) tissue. Among the circRNA expression profile, two circRNAs (hsa_circ_0014130 and hsa_circ_0016760) were selected for validation in ten pairs of NSCLC and adjacent non-cancerous tissues by real-time quantitative reverse transcription-polymerase chain reaction (qRT-PCR). Only hsa_circ_0014130 exhibited significantly overexpressed in NSCLC tissues (*P* < 0.001), which were further confirmed in another 36 matched tissue samples using qRT-PCR. Hsa_circ_0014130 expression significantly correlated with TNM stage (*P* = 0.001) and lymphatic metastasis (*P* = 0.004). The area under the receiver operating characteristic curve was 0.878 (95% confidence interval = 0.804–0.951; *P* < 0.001), which showed good diagnostic potential. Bioinformatics platforms predicted that hsa_circ_0014130 might interact with five miRNAs and their corresponding mRNAs. Gene oncology analysis and pathway analysis revealed that hsa_circ_0014130 could participate in NSCLC development. In summary, our findings indicated that hsa_circ_0014130 could be used as a potential NSCLC biomarker and might be closely related to the carcinogenesis of NSCLC.

## Introduction

Lung cancer is the leading cause of cancer death among males in both more and less developed countries, and has surpassed breast cancer as the leading cause of cancer death among females in more developed countries^1^. Non-small cell lung cancer (NSCLC) accounts for more than 80% of all lung cancer cases, consists of squamous cell carcinoma, large cell carcinomas and adenocarcinoma, and most patients with NSCLC have advanced local invasion and/or distant metastases at the time of diagnosis^2^. Therefore, better understanding of the molecular mechanisms associated with NSCLC pathogenesis is critical to the development of effective diagnostic and therapeutic approaches, which in turn contributes to identify novel biomarkers for NSCLC detection and treatment targets, and even to develop personalized therapies for individual patients NSCLC with in the future.

Circular RNAs (circRNAs) are a unique class of endogenous noncoding RNAs (ncRNAs) that, unlike linear RNA, form a closed continuous loop by back-splicing with covalently joined 3′- and 5′-ends^3^. As a result of this closed structure, circRNAs have been shown to be highly stable and largely resistant to RNA degradative pathways^4^, which highlights clear advantages in using circRNAs as novel molecular biomarkers for many diseases. Many studies have reported that circRNAs have numerous biologic functions, which are involved in forming RNA-protein complexes, acting as microRNA (miRNA) sponges, and regulating targeted gene transcription and splicing^5^. Therefore, it has been hypothesized that circRNAs potentially regulate disease progression by sequestering a miRNA associated with a particular disease^6^. Recent studies indicated that circRNAs might play an important role in carcinogenesis and tumor progression^7,8^. Some studies have revealed that circRNAs exhibit dysregulated expression in human various cancers including esophageal squamous cell carcinoma, gastric cancer, hepatocellular carcinoma, colorectal cancer, laryngeal cancer, gliomas^9–15^ and so on. It has been reported that circRNA might serve as a novel potential biomarker for cancer diagnosis^16^. However, the role of circRNAs in NSCLC has not been well studied. A report has showed that circular RNA-ITCH may compete with ITCH to bind to miR-7 and miR-214 and may be involved in lung cancer development^17^. Hsa_circ_100876 has been suggested as a potential prognostic biomarker for NSCLC^18^. Hsa_circ_0013958 has been indicated to be used as a potential non-invasive biomarker for the early detection and screening of lung adenocarcinoma^19^. Despite this potential link with circRNAs, the global circRNA expression profile in NSCLC has not been fully uncovered.

Hence, in the present study we first performed circRNA microarray to investigate the differential expression profiles of circRNAs between NSCLC tissues and paired adjacent non-cancerous tissues. Subsequently, from these differentially expressed circRNAs detected in microarray, two circRNAs (hsa_circ_0014130 and hsa_circ_0016760) were confirmed by real-time quantitative reverse transcription-polymerase chain reaction (qRT-PCR). We then selected hsa_circ_0014130, with significantly upregulated expression in the validation test above, as a targeted circRNA to further explore its clinical significance and application in NSCLC. In addition, a bioinformatics analysis was used to predict the miRNA binding sites and the related mRNAs of hsa_circ_0014130. These results indicate that the upregulated expression of hsa_circ_0014130 significantly associates with some clinicopathological factors of NSCLC patients, and may serve as a novel potential tumor marker and therapeutic target for NSCLC.

## Results

### Validation of RNA quality

Total RNA was extracted from each sample, and the results of RNA quality and RNA integrity were displayed in Supplementary Figure 1.

### Differential circRNA expression profiles in NSCLC

**Figure 1:**
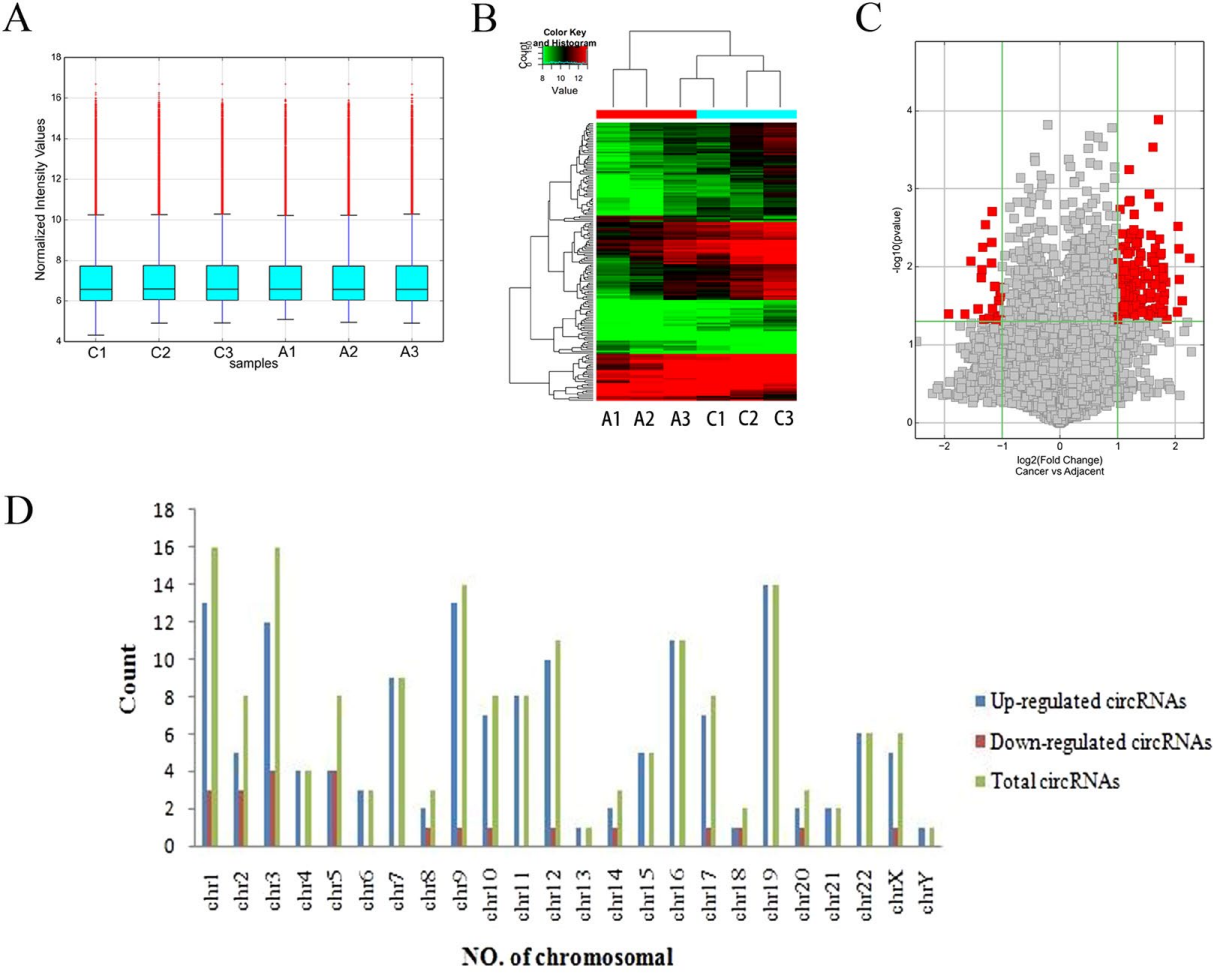
Differences and characterizations of circRNA expression profile between NSCLC tissues and paired adjacent non-cancerous tissues. (**A**) Box plots show the distribution of circRNAs for the six samples (C for NSCLC and A for adjacent non-cancerous tissues). The distributions were nearly the same after normalization. (**B**) Unsupervised hierarchical clustering shows a distinguishable circRNA expression profiling among the six samples (C for NSCLC and A for adjacent non-cancerous tissues). Each column represents the expression profile of a tissue sample, and each row corresponds to a circRNA. “Red” indicates higher expression level, and “green” indicates lower expression level. (**C**) Volcano plots are used to visualize the differential circRNA expression. The vertical green lines correspond to 2.0-fold up and down, and the horizontal green line represents a *P*-value of 0.05. The red points in plot represent the differentially expressed circRNAs with statistical significance. (**D**) Chromosomal distributions of differentially expressed circRNAs.

Using high-throughput human circRNA microarray, we assessed the differences of circRNA expression profiles between NSCLC tissues and paired adjacent non-cancerous tissues from 3 patients. We drew a Box plot to quickly visualize the distribution of the intensities from all the datasets after normalization and found that the distribution of log2 ratios was similar in all the tested samples (Fig. 1A). Cluster analysis segregated samples into groups based on differences in their expression levels, and hypothetic relationships among the samples. The results of hierarchical clustering showed distinguishable circRNA expression profiling among 6 samples (Fig. 1B). These data indicated that circRNAs have a different expression pattern in NSCLC compared with that in adjacent lung tissues. The Volcano plot was performed to visualize the significant differences (fold change > 2.0, *P* value < 0.05) between NSCLC and non-cancerous tissues (Fig. 1C). Moreover, the distributions of differentially expressed circRNAs in human chromosomes showed that most circRNAs were transcribed from chr1, chr3, chr9, chr12, chr16 and chr19, but seldom from chr13, chr21 and chrY (Fig. 1D). The microarray data showed 171 circRNAs were found to be differentially expressed in NSCLC tissues (fold change > 2.0, *P* value < 0.05). Among them, 148 circRNAs were upregulated and 23 were downregulated in tumor tissues. The top 5 upregulated and downregulated circRNAs were presented in Table 1.

**Table 1:** Biological information regarding the top 5 upregulated and downregulated circRNAs.

CircRNA ID	Fold change	*P*-value	circRNA type	Chromosome	Best transcript*	Gene symbol
**Upregulated**						
hsa_circ_0000735	4.75	0.00774	exonic	chr17	NM_002558	P2RX1
hsa_circ_0016760	4.36	0.02712	exonic	chr1	NM_053052	SNAP47
hsa_circ_0003645	4.17	0.00586	exonic	chr16	NM_020314	C16orf62
hsa_circ_0087862	4.13	0.00301	exonic	chr9	NM_002874	RAD23B
hsa_circ_0026134	4.10	0.03763	exonic	chr12	uc001rtt.1	TUBA1C
**Downregulated**						
hsa_circ_0005730	3.12	0.04028	exonic	chr5	NM_001799	CDK7
hsa_circ_0091000	2.91	0.00842	exonic	chrX	NM_014878	NONO
hsa_circ_0014130	2.55	0.01090	exonic	chr1	NM_003557	PIP5K1A
hsa_circ_0071989	2.49	0.04684	exonic	chr5	NM_012334	MYO10
hsa_circ_0092368	2.31	0.04560	intronic	chr1	ENST00000361427	HMGN2

*Best transcript, is transcribed from the same gene position with circular RNA, the sequence information is most similar to circular RNA.

### Amplification of hsa_circ_0014130 and hsa_circ_0016760

**Figure 2:**
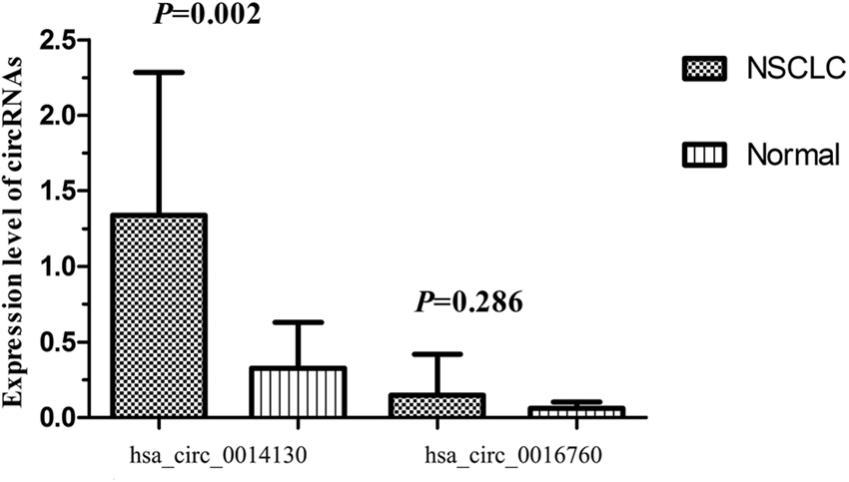
The expression levels of candidate circRNAs were validated by performing RT-qPCR (in triplicate) with ten pairs of NSCLC tissues and adjacent lung tissues. RNA expression levels were normalized to expression of the reference gene β-actin, only hsa_circ_0014130 was significantly differentially expressed between the 2 groups (4.06-fold, *P* = 0.002).

We used qRT-PCR assays to verify 2 typically differential expression circRNAs (hsa_circ_0014130 and hsa_circ_0016760) in 10 samples of NSCLC tissues and their paired adjacent lung tissues. The primers were specifically capable of amplifying the backsplice sites of each circRNA, and the PCR results were validated by melt-curve analysis (Supplementary Fig. 2). These tests confirmed that hsa_circ_0014130 and hsa_circ_0016760 existed in NSCLC tissues and could be specifically amplified by qRT-PCR. As shown in Fig. 2, we found that the hsa_circ_0014130 expression levels in NSCLC tissues were significantly higher than those in corresponding non-cancerous tissues, but hsa_circ_0016760 expression levels had no significant difference between the NSCLC tissues and the corresponding non-cancerous tissues. However, the expression level of hsa_circ_0014130 was not consistent with that measured by microarray analysis, in which the hsa_circ_0014130 expression was significantly downregulated (Table 1).

### Upregulation of hsa_circ_0014130 expression in NSCLC Tissues

**Figure 3:**
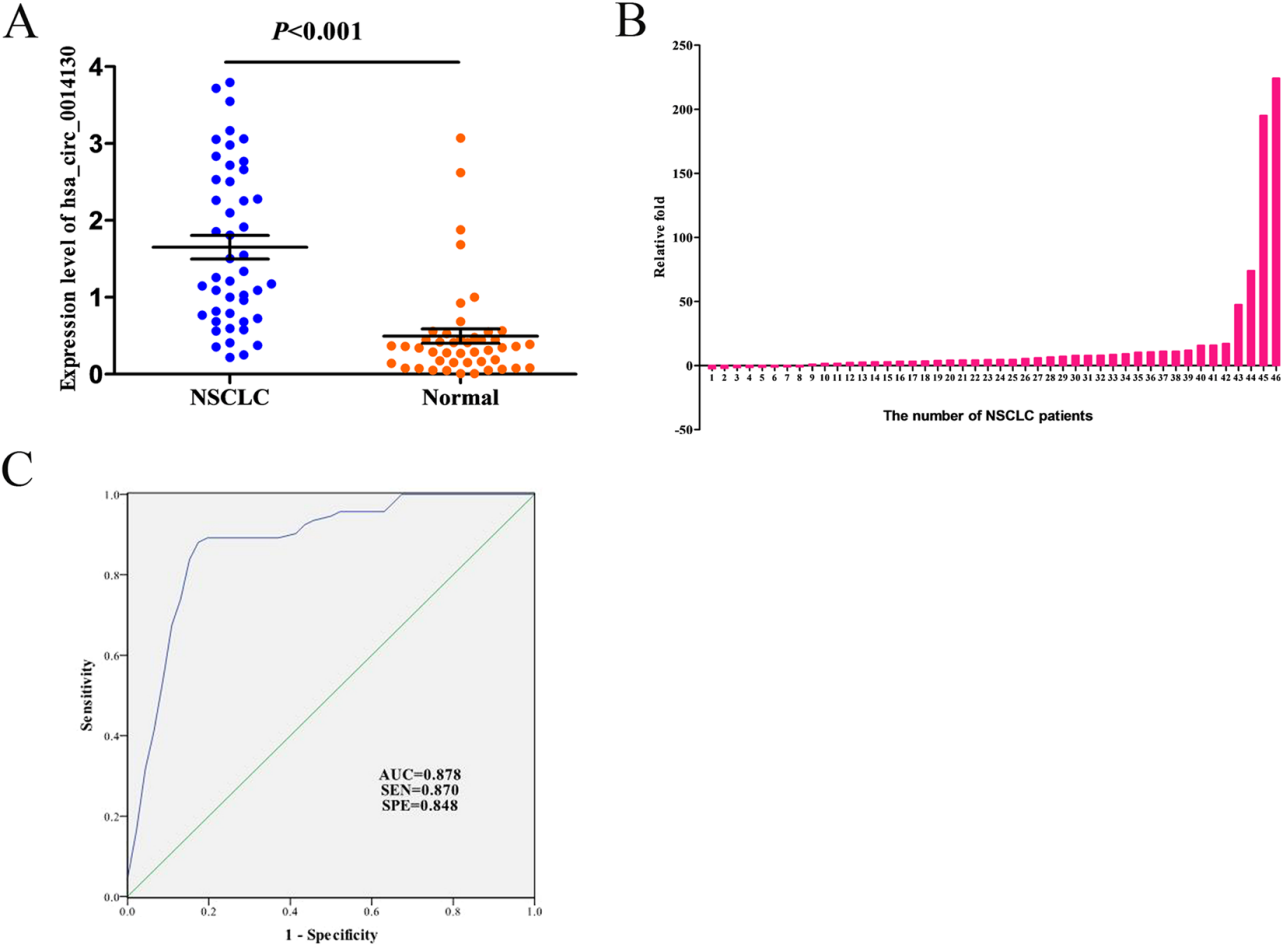
Hsa_circ_0014130 significantly upregulated in cancer tissues could serve as a biomarker for NSCLC. (**A**) The expression levels of hsa_circ_0014130 in the NSCLC group are significantly higher than those in corresponding non-cancerous tissues (n = 46) (*P* < 0.001). (**B**) Hsa_circ_0014130 levels were significantly upregulated in 82.6% (38/46) of NSCLC tissues. (**C**) Receiver operating characteristic (ROC) curve of hsa_circ_0014130 was built for differentiating NSCLC tissues from controls. The area under curve was 0.878. The cutoff value was 0.573, and the sensitivity and specificity were 87.0% and 84.8%, respectively (*P* < 0.001).

By means of inquiring from circBase (http://www.circbase.org), we knew that hsa_circ_0014130 was located at chromosome 1q21.3 and was composed of five exons (Supplementary Fig. 3A). The PCR results of hsa_circ_0014130 were validated by gel electrophoresis (Supplementary Fig. 3B) and sequencing the amplified product (Supplementary Fig. 3C), which showed that the primers were specifically capable of amplifying the back splice sites of hsa_circ_0014130. Next, to validate the previous study results, we expanded the sample size. The expression levels of hsa_circ_0014130 in 46 NSCLC tissues and their matched adjacent non-cancerous tissues were measured by qRT-PCR method, which showed that hsa_circ_0014130 expression was significantly upregulated in NSCLC tissues (*P < *0.001, Fig. 3A). Moreover, the results showed that hsa_circ_0014130 expression was significantly upregulated in 82.6% (38/46) NSCLC tissues compared with the adjacent non-cancerous tissues (Fig. 3B). We then used the receiver-operating characteristic (ROC) curve to investigate the diagnostic value of hsa_circ_0014130 in distinguishing NSCLC tissues from adjacent non-cancerous tissues. When the expression level of hsa_circ_0014130 was analyzed for this purpose, the area under the ROC curve (AUC) was 0.878 (Fig. 3C), and the optimal cutoff value of hsa_circ_0014130 was 0.573, with sensitivity and specificity were 87.0% and 84.8%, respectively.

Furthermore, we aimed to determine whether a high level of hsa_circ_0014130 in patients was associated with clinicopathological parameters. As shown in Table 2, we found that the level of hsa_circ_0014130 expression showed no significant differences between groups of different genders (*P* = 0.513), ages (*P* = 0.239), smoking/ nonsmoking (*P* = 0.748), tumor sizes (*P = *0.074) or pathologic types (P = 0.381). However, there were significant associations between the level of hsa_circ_0014130 expression and TNM stage (*P* = 0.001) or lymphatic metastasis (*P* = 0.004).

**Table 2:** The Associations between the hsa_circ_0014130 expression level and clinicopathological characteristics of patients with NSCLC.

Characteristics	NO. of patiens (%)	hsa_circ_0014130 (Mean ± SD)	*P* value
Gender			
Female	11 (23.92)	1.833 ± 1.049	0.513
Male	35 (76.08)	1.595 ± 1.042	
Age (years)			
≥ 60	36 (78.26)	1.556 ± 1.076	0.239
<60	10 (21.74)	1.997 ± 0.841	
Smoking			
Yes	29 (63.04)	1.690 ± 1.010	0.748
No	17 (36.96)	1.587 ± 1.111	
Tumor diameter			
≥ 5	19 (41.30)	1.978 ± 0.940	0.074
<5	27 (58.70)	1.423 ± 1.058	
Histological subtype			
Adenocarcinoma	31 (67.39)	1.558 ± 1.094	0.381
Squamous cell	15 (32.61)	1.847 ± 0.914	
TNM stage			
I	20 (43.48)	1.063 ± 0.677	0.001*
II	16 (34.78)	1.921 ± 0.995	
III	10 (21.74)	2.401 ± 1.114	
Lymphatic metastasis			
Yes	14 (30.43)	2.293 ± 1.090	0.004*
No	32 (69.57)	1.372 ± 0.893	

*P <0.05, compared among different groups. The expression level of hsa_circ_0014130 was significantly associated with TNM stage and lymphatic metastasis.

### CircRNA-microRNA-mRNA co-expression network for the hsa_circ_0014130

**Figure 4:**
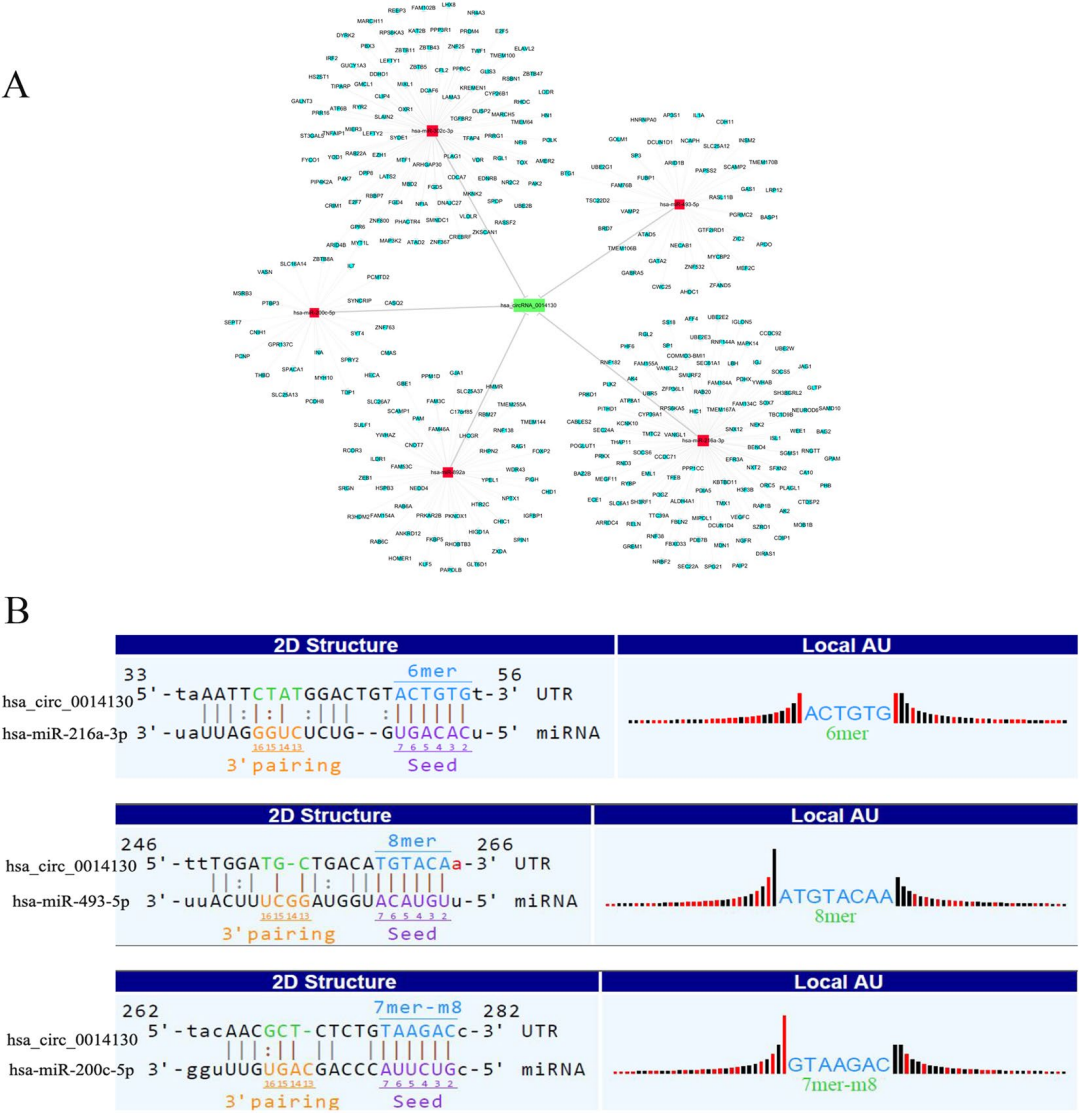
The predicted hsa_circ_0014130 targeted circRNA-miRNA-mRNA gene co-expression network based on sequence-pairing prediction. (**A**) The co-expression network was drawn with the cytoscape software. Five miRNAs and their mRNA target genes were found with overlapping results. As shown in this figure, miR-216a-3p exhibited the largest interaction network followed by miR-302a-3p, miR-892a, miR-493-5p, and miR-200c-5p. (**B**) Seed sequence matching predicted the direct interaction of hsa_circRNA_0014130 with the three cancer-related miRNAs: miR-216a-3p, miR-493-5p and miR-200c-5p.

As circRNA and miRNA sequences were aligned, and circRNAs interacted with miRNAs via miRNA response elements (MREs), 5 miRNAs with the highest mirSVR scores were identified for each differentially expressed circRNA using miRNA target-prediction software. Predicted Top-5 miRNAs regarding the top 5 upregulated and downregulated circRNAs including hsa_circ_0014130 were shown in Table 3. We assumed that hsa_circ_0014130 act as a miRNA sponge to regulate its circRNA-miRNA-mRNA network. We then predicted the target genes of Top-5 miRNAs by means of targetscan7.1 and mirdbV5. We generally accepted the overlapping results of two databases, and there were 333 target genes for hsa_circ_0014130 (Supplementary Fig. 4). Based on these analysis results, a total of 5 miRNAs and 333 mRNAs were predicted to have an interaction with hsa_circ_0014130 in this study. Cytoscape analysis of the circRNA-miRNA-mRNA interaction network of hsa_circ_0014130 indicated that miR-216a-3p exhibited the largest interaction network followed by miR-302a-3p, miR-892a, miR-493-5p, and miR-200c-5p (Fig. 4A). Since predicted target miRNAs including miR-216a-3p, miR-493-5p and miR-200c-5p, proved to be downregulated in cancer progression in previous research results, we utilized the public databases [circBase (http://www.circbase.org)] to screen for targeted miRNAs and the results based on seed sequence matching and specific base pairing displayed the three cancer-related miRNAs: miR-216a-3p, miR-493-5p and miR-200c-5p, had a binding site for hsa_circ_0014130 (Fig. 4B).

**Table 3:** Predicted miRNA response elements regarding the top 5 upregulated and downregulated circRNAs.

CircRNA ID	Predicted miRNA response elements (MREs)				
	MRE1	MRE2	MRE3	MRE4	MRE5
**Upregulated**					
hsa_circ_0000735	hsa-miR-5787	hsa-miR-3189-3p	hsa-miR-762	hsa-miR-6791-5p	hsa-miR-3157-5p
hsa_circ_0016760	hsa-miR-646	hsa-miR-625-5p	hsa-miR-29b-1-5p	hsa-miR-129-5p	hsa-miR-424-5p
hsa_circ_0003645	hsa-miR-1301-3p	hsa-miR-422a	hsa-miR-378a-3p	hsa-miR-602	hsa-miR-378d
hsa_circ_0087862	hsa-miR-325	hsa-miR-593-3p	hsa-miR-512-3p	hsa-miR-653-5p	hsa-miR-766-5p
hsa_circ_0026134	hsa-miR-593-5p	hsa-miR-4739	hsa-miR-1293	hsa-miR-548al	hsa-miR-103a-3p
**Downregulated**					
hsa_circ_0005730	hsa-let-7f-2-3p	hsa-miR-382-5p	hsa-miR-487a-5p	hsa-miR-519d-5p	hsa-miR-22-3p
hsa_circ_0091000	hsa-miR-197-5p	hsa-miR-103a-2-5p	hsa-miR-4778-3p	hsa-miR-7851-3p	hsa-miR-4436b-5p
hsa_circ_0014130	hsa-miR-892a	hsa-miR-216a-3p	hsa-miR-302c-3p	hsa-miR-493-5p	hsa-miR-200c-5p
hsa_circ_0071989	hsa-miR-877-3p	hsa-miR-103a-2-5p	hsa-miR-432-5p	hsa-miR-338-3p	hsa-miR-370-3p
hsa_circ_0092368	hsa-let-7e-5p	hsa-miR-330-5p	hsa-miR-326	hsa-miR-580-3p	hsa-miR-769-5p

### Bioinformatics analysis of the predicted network genes for the hsa_circ_0014130

**Figure 5:**
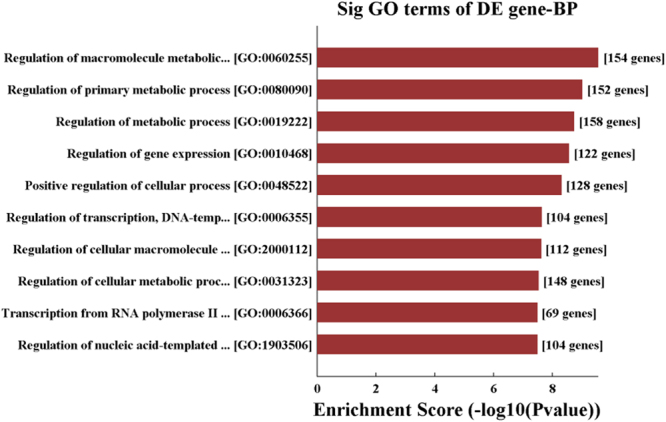
Gene ontology (GO) enrichment analysis for hsa_circ_0014130 in terms of biological processes (BP). The top 10 significantly enriched target genes and their scores (negative logarithm of P value) are listed as the x-axis and the y-axis, respectively. The horizontal axis represents the significant level of GOs.

**Figure 6:**
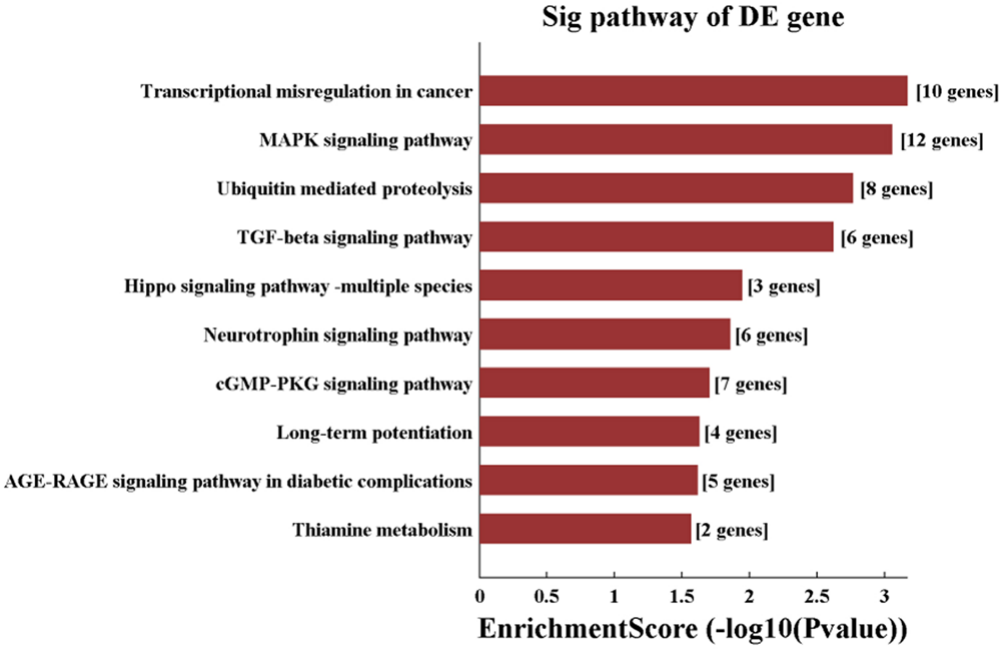
KEGG pathway analysis for hsa_circ_0014130*. The top 10 significantly enriched pathways and their scores (negative logarithm of P value) were listed as the x-axis and the y-axis, respectively. The horizontal axis represented the significant level of pathways. *This image was obtained from KEGG (http://www.kegg.jp/kegg/kegg1.html).

Gene Ontology (GO) analysis for hsa_circ_0014130 was performed to explore the functional roles of the top 10 significantly enriched target genes in terms of biological processes (BP) (Fig. 5), cellular components (CC) and molecular functions (MF) (Supplementary Fig. 5). From these results, we found that hsa_circ_0014130 showed a strong relationship with the regulation of metabolic process, the intracellular membrane-bounded organelle, DNA binding and its regulation of transcription, and so on. Kyoto Encyclopedia of Genes and Genomes (KEGG) analysis for hsa_circ_0014130 showed that there were the top 10 significantly enriched pathways including Transcriptional dysregulation in cancer, MAPK signaling pathway, Ubiquitin mediated proteolysis, TGF−beta signaling pathway and so on (Fig. 6). Especially, there were 12 target genes enrichment in MAPK signaling pathway (Supplementary Fig. 6). These processes and pathways were associated with human tumorigenesis and metastasis.

## Discussion

In recent years, with the rapid development and widespread application of RNA sequencing, researchers have found that many exonic transcripts can form circRNAs through non-linear reverse splicing or gene rearrangement. Moreover, they account for a large proportion of all spliced transcripts^20^. CircRNAs may arise from exons or introns^21^. An increasing number of researchers have begun to study potential functions of circRNAs in all kinds of diseases such as nervous system, cardiovascular system diseases and cancers^7,8,22,23^. Some altered circRNAs have been demonstrated to associate with tumor development, invasion, metastasis or patient prognosis^9–15^. Compared with other ncRNAs such as miRNAs and long noncoding RNAs (lncRNAs), circRNAs are highly conserved sequences and high degree of stability in mammalian cells^4^, these properties provide circRNAs with the potential to become ideal biomarkers for diagnosis of disease, including cancers^16^ and potential treatment target. Currently, although three circRNAs including cir-ITCH, hsa_circ_100876, hsa_circ_0013958 were studied in lung cancer^17–19^, the clinical value and role of circRNAs in NSCLC remained largely unknown.

In this study, we first investigated circRNA expression profile in human NSCLC by high-throughput circRNA microarray. Our results showed that circRNA expression in NSCLC tissues(n = 3) is significantly different from that in adjacent non-cancerous tissues (n = 3) (Fig. 1). Compared with non-cancerous tissues, our microarray data showed a total of 148 circRNAs were significantly upregulated and 23 were significantly downregulated in NSCLC tissues. Moreover, the distributions of differentially expressed circRNAs in human chromosomes showed that most circRNAs were transcribed from chr1, chr3, chr9, chr12, chr16 and chr19 (Fig. 1D). After validating the expression of 2 circRNAs (hsa_circ_0014130 and hsa_circ_0016760) in additional tissue samples (n = 10), only hsa_circ_0014130 was confirmed to be significantly upregulated in NSCLC (P = 0.002) (Fig. 2). However, the expression level of hsa_circ_0014130 was not consistent with that measured by microarray analysis, in which the hsa_circ_0014130 expression was significantly downregulated (Table 1). These results indicated that the validation of differentially expressed circRNAs in microarray analysis was an essential step of this screening study. Furthermore, to validate the study results, we expanded the sample size to detect the expression of hsa_circ_0014130 in additional tissue samples (n = 36). Our results showed that hsa_circ_0014130 was significantly upregulated in 82.6% (38/46) NSCLC tissues with average 3.34 fold compared with their adjacent non-cancerous tissues, and the AUC of hsa_circ_0014130 was up to 0.878 (Fig. 3). The ROC analyses confirmed that hsa_circ_0014130 had a high degree of specificity and sensitivity. More importantly, considering clinicopathological factors, we found that high expression levels of hsa_circ_0014130 in NSCLC were significantly associated with TNM stage and lymph node metastasis (Table 2), which are crucial factors in the evaluation of the prognosis of NSCLC. Taken together, these findings indicated that the circRNA 0014130 might be involved in carcinogenesis, progress and metastasis of NSCLC, might be used as a potential non-invasive biomarker of NSCLC and a new target for treatment of NSCLC.

CircRNAs represent a class of widespread ncRNAs, since they are capable of negatively regulating the effects of miRNAs upon their target mRNAs by directly competing for binding to shared miRNAs via MREs, thereby essentially functioning as ‘miRNA sponges’ within the cellular RNA network^24,25^. Compared with linear miRNA sponges, circRNAs have more miRNA binding sites and higher expression levels, and may be more effective in sequestering miRNAs^26,27^. Increasing evidence has indicated that the aberrant expression of circRNAs may promote cancer pathogenesis by absorbing cancer-associated miRNAs^7^. In the present study, to further understand the biological function of hsa_circ_0014130, the top 5 miRNAs with the highest mirSVRs were identified (including miR-216a-3p, miR-302a-3p, miR-892a, miR-493-5p and miR-200c-5p) (Table 3), and the hsa_circ_0014130-miRNA-gene network was predicted using TargetScan and miRanda. In essence, this network map diagramed a cellular RNA network consisting of hsa_circ_0014130 interacting with 5 miRNA nodes and 333 target genes (Fig. 4A). By searching the literatures from PubMed (https://www.ncbi.nlm.nih.gov/pubmed/), before Octomber 26, 2017, miR-216a-3p, miR-493-5p and miR-200c-5p were proved to be downregulated in cancer progression in previous research results. According to the prediction results of circBase^28^, we found that miR-216a-3p, miR-493-5p and miR-200c-5p might interact with hsa_circ_0014130 (Fig. 4B). As hsa_circ_0014130 might function as a potent miRNA sponge, we considered the regulatory network in which the three cancer-related miRNAs: miR-216a-3p, miR-493-5p and miR-200c-5p could participate.

It have been found that miR-216a-3p was significantly reduced in pancreatic ductal adenocarcinoma and could affect colorectal cancer cell proliferation by inhibiting COX-2 and ALOX5 expression^29,30^. miR-493-5p has been found to be deregulated in several tumours, such as breast cancer and myeloid leukemia^31,32^. It has been reported that the expression level of miR-493-5p was down-regulated in NSCLC patients and the overexpression of miR-493-5p significantly inhibited NSCLC cell proliferative capacity by suppressing the expression of oncogene ITGB1^33^. Similar to miR-216a-3p and miR-493-5p, miR-200c-5p was reportedly downregulated in human hepatocellular carcinoma (HCC) and replenishing of miR-200c-5p suppressed the proliferation, migration and invasion of HCC cells by suppressing MAD2L1^34^. Therefore, the decreased expression and inhibited function of miR-216a-3p, miR-493-5p and miR-200c-5p in cancer further support our hypothesis that hsa_circ_0014130 functions as a miRNA sponge to regulate the more comprehensive hsa_circ_0014130-miRNA-mRNA network.

In this study, we found that a large number of mRNAs might take part in this hsa_circ_0014130-miRNA-gene network described above, such as interleukin 7, insulin-like growth factor binding protein 1, transcription factor E2F7 and so on. So we functionally examined the target genes using GO and KEGG pathway analysis. GO enrichment analysis (Fig. 5) revealed that target genes were involved in the regulation of crucial metabolic processes and the transcription regulation of DNA binding, indicating that regulating these genes in the cellular response is of great importance during the development of NSCLC. Among the KEGG pathways (Fig. 6) found in this study, MAPK signaling pathway has been reported to be a key mediator of the growth, invasion, and migration of NSCLC cells^35^. Ubiquitin mediated proteolysis pathway has been found to promote its ubiquitin-proteasome-dependent degradation and consequently lead to radiation-induced apoptosis in NSCLC cells^36^. Since MAPK signaling pathway and ubiquitin mediated proteolysis pathway are strongly associated with NSCLC cell proliferation, invasion, and metastasis, we speculate that the hsa_circ_0014130-microRNA- mRNA axis may be the possible mechanism promoting the development of NSCLC, and it is worthwhile to further investigate the over-expressed hsa_circ_0014130 as an inhibitor of miRNA and its potential mechanism.

In conclusion, the present study revealed the expression profile of circRNAs in NSCLC tissues and demonstrated that circRNAs were aberrantly expressed in NSCLC. Our study validated the significant upregulation of hsa_circ_0014130 and analyzed the relationship of between this circRNA and clinical features in NSCLC patients, suggesting its potential involvement in NSCLC tumorigenesis and its potential use as a novel biomarker for NSCLC diagnosis. In the future, it’s necessary to explore the detailed molecular mechanisms by which hsa_circ_0014130 functions as miRNA sponges to regulate NSCLC occurrence and development.

## Methods

### Patients and specimens

The study included patients with NSCLC who underwent partial or total pulmonary lobectomy at the Department of Thoracic Surgery of Beijing Anzhen Hospital of Capital Medical University (China) between September 2016 and September 2017. NSCLC tissues were collected from the tumor surface, and the corresponding adjacent non-cancerous tissues were taken 5 cm from the edge of the cancer and contained no obvious tumor cells. After removal from the body, the specimens were preserved in liquid nitrogen within 5 minutes of excision. They were transported frozen to the laboratory for storage at −80 °C until use. For excluding confounding factors affecting the steady of patients’ circRNA profiling, none of the enrolled patients received chemotherapy, radiotherapy, or target therapy before they underwent surgery. All cases were diagnosed as NSCLC by two independent pathologists. Tumor histological grading and staging were based on the World Health Organization classification criteria and the seventh edition of the International Union against Cancer Tumor Node Metastasis (TNM) staging system. In total, 46 NSCLC tissues and 46 adjacent lung tissues were obtained from 46 patients (11 females and 35 males). For circRNA microarray analysis, 3 NSCLC tissues and corresponding 3 adjacent lung tissues were randomly selected. And other 46 NSCLC tissues and corresponding 46 adjacent tissues were prepared for qRT-PCR validation experiments. We obtained written informed consent from all patients. The study was approved by the Medical Ethics Commission of Capital Medical University. The study was in accordance with the provisions of Medical Ethics Commission of Capital Medical University. I confirmed that all methods were performed in accordance with the relevant guidelines and regulations.

### Total RNA isolation and quality control (QC)

Total RNA was isolated from tumor and adjacent tissues using the TRIzol reagent (Invitrogen, Carlsbad, CA, USA) following the manufacturer’s protocol. The purity and concentration of RNA samples were examined spectrophotometrically by absorbance measurements at 260 nm, 280 nm and 230 nm using the NanoDrop ND-1000 (Thermo Fisher Scientific, Wilmington, DE, USA). Specifically, OD260/OD280 ratios between 1.8 and 2.1 were deemed acceptable, while OD260/OD230 ratios of greater than 1.8 were deemed acceptable. RNA integrity and contamination was tested by denaturing agarose gel electrophoresis. RNA was prepared and stored at −80 °C for the validation experiments.

### Sample Preparation and Microarray Detection

Total RNA from 6 samples (3 NSCLC samples and 3 paired adjacent non-cancerous samples) was treated with Rnase R (Epicentre, Madison, WI, USA) to remove linear RNAs and to enrich circRNAs. Then, the enriched circRNAs were amplified and transcribed into fluorescent circRNA utilizing a random priming method according to Arraystar Super RNA Labeling Kit’s instructions (Arraystar, Rockville, MD, USA). The labeled circRNAs were purified by RNeasy Mini Kit (Qiagen, Germany). The concentration and specific activity of the labeled circRNAs (pmol Cy3/μg circRNAs) were measured by NanoDrop ND-1000. The labeled circRNAs were hybridized onto the Arraystar Human circRNA Arrays (8 × 15 K, Arraystar). The slides were incubated for 17 h at 65 °C in an Agilent Hybridization Oven (Agilent Technologies, Santa Clara, CA, USA). The hybridized microarrays were washed, fixed, and scanned with an Agilent G2505C Scanner (Agilent Technologies, Santa Clara, CA, USA).

### CircRNA Microarray Data Analysis

Scanned images were imported into Agilent Feature Extraction software (version 11.0.1.1, Agilent) for raw data extraction. Following quantile normalization of the data using a log2 ratio (R software package, version 3.1.2), we performed a low-intensity filtering. The circRNAs with at least 3 out of 6 samples flagged in “P” or “M” (“all targets value”) were retained for further analysis. The Box Plot was drawn to visualize the distribution of the intensities from all datasets after normalization. To determine whether circRNA profiles were informative with regards to tissue type, an unsupervised, hierarchical cluster analysis was conducted based on the circRNA expression levels in 3 NSCLC specimens and adjacent lung tissues. Then, fold-change filtering and Student’s *t*-testing were applied to identify differential circRNA between NSCLC tumors versus matching adjacent lung tissues. Specifically, only the circRNAs that exhibited fold-changes of greater than 2.0 and Student’s *t*-test *p*-values of less than 0.05 were identified as significantly differential circRNAs. Volcano plot was used to visualize the significantly differential circRNAs between between NSCLC and non-cancerous tissues.

### qRT-PCR validation of Candidate circRNAs

To validate the expression profiles of circRNAs in three matched pairs of NSCLC and adjacent specimens, considering the group raw signal intensity (>500.0), the significant difference of raw signal intensity (>100.0), the fold change of expression(FC >2.0), and that circRNAs whose target miRNAs predicted in the microarray analysis proved to be in related with cancer progression in previous research results, 2 circRNAs (hsa_circ_0014130 and hsa_circ_0016760) were selected for validation study by qRT-PCR as follows. After RNA extraction from NSCLC tumors and matching adjacent lung tissues, cDNA was synthesized with a reverse transcriptase according to the kit’s instructions (SuperScript First-Strand Synthesis System for RT-PCR, Invitrogen, Carlsbad, CA, USA). Divergent primers of hsa_circ_0014130 and hsa_circ_0016760 were designed with Primer Premier software version 5.0 (Premier Biosoft International, Palo Alto, CA, USA), ensuring that circRNAs were amplified through head-to-tail splicing^4^. β-actin, a housekeeping gene, was used as internal standard for normalization. Primers were synthesized by Bioligo Biotech (Shanghai, China). The sequences of hsa_circ_0014130, hsa_circ_0016760 and β-actin primers were as follows: 5′ AGATTCCCTAACCTCAACCAGA3′ (forward) and 5′ CGAATGTTCTTGCCACCTGC3′ (reverse) for hsa_circ_0014130; 5′ TGCATTGGTGCTCAGAAGCG3′ (forward) and 5′ TCTGTTCCTGGGTCTGTGTGC3′ (reverse) for hsa_circ_0016760; and 5′ GTGGCCGAGGACTTTGATTG3′ (forward) and 5′ CCTGTAACAACGCATCTCATATT3′ (reverse) for β-actin. PCR was conducted in a 10-μl reaction volume consisting of the following: 2.0 μl cDNA, 5 μl 2 × PCR Master mix (PCR Master Mix, Arraystar, Rockville, MD, USA), 0.5 μl primer forward (5 μM), 0.5 μl primer reverse (5 μM), and 2.0 μl H_2_O. The qPCR reaction was performed on an ABI QuantStudio^TM^ 5 System (Applied Biosystems, DE, USA) as follows: initial denaturation at 95 °C for 10 min, 40 cycles of amplification at 95 °C for 10 sec, annealing and extension at 60 °C for 1 min. Amplification products were analyzed by 1.5% agarose gel electrophoresis, stained with 1∶10000 dilution of GelRed Nucleic Acid gel stain (Biotium, CA, USA) and visualized under ultraviolet illumination for band size consistency. All the experiments were conducted in triplicate. The data were analyzed by using the comparative cycle threshold (2^−ΔΔCt^) ^19^ to represent a relative expression level of circRNAs.

### Cloning and sequencing of qRT-PCR products

Two qRT-PCR products of hsa_circ_0014130 in NSCLC tumor and adjacent lung tissue were purified using PCR Product Purification Kit (Ensurebio Biotech Co., Ltd., Shanghai, China), and then cloned using PMD18-T Vector Cloning Kit (Takara Bio Inc., Dalian, China) following the manufacturer’s instructions. DNA sequencing was performed by Life Technologies (Thermo Fisher Scientific, Wilmington, DE, USA).

### Prediction of circRNA-miRNA-mRNA co-expression network for the hsa_circ_0014130

The homemade computer program of Arraystar (Rockville, MD, USA) based on TargetScan^37^ and miRanda^38^ was applied to predict miRNA targets of circRNAs and the circRNA/miRNA interaction. To focus the targeted miRNA profile, the miRNA support vector regression (mirSVR) algorithm was used to score and rank the efficiency of the predicted miRNA targets^39^. Accordingly, for each circRNA, we identified 5 miRNAs with the highest mirSVR score to establish a “Top-5” circRNA-miRNA network (1 circRNA connecting to 5 miRNAs). To further elucidate correlations between circRNAs and miRNA, Cytoscape 3.01 was then used to diagram the potential map of the circRNA/miRNA/mRNA interaction network of hsa_circ_0014130. The size of each node in the network map represents the number of putative miRNA functionally connected to hsa_circ_0014130.

### Prediction of circRNA-miRNA-target gene associations for the hsa_circ_0014130

To gain further insights into the functions of hsa_circ_0014130, putative target genes of miRNAs were predicted by two databases including targetscan7.1 (http://www.targetscan.org/vert_71/) and mirdbV5 (http://mirdb.org/miRDB/). GO analysis was performed to explore the functional roles of target genes in terms of biological processes, cellular components and molecular functions. Biological pathways defined by KEGG^40^, Biocarta and Reactome (http://www.genome.jp/kegg/) were identified by Database for Annotation, Visualization and Integrated Discovery (DAVID; http://www.david.abcc.ncifcrf.gov/). The predicted gene functions of the hsa_circ_0014130 in the circRNA-miRNA-target gene associations were annotated using GO and KEGG pathway analysis.

### Statistical Analysis

All results are expressed as the mean ± SD. All statistical data were analyzed using Statistical Program for Social Sciences (SPSS) 19.0 software (SPSS, Chicago, IL, USA). GraphPad Prism 5.0 (GraphPad Software, La Jolla, CA) was used to plot all graphs. The significance of qRT-PCR validation between the NSCLC tissue group and the adjacent tissue group was analyzed by the Student *t* test for paired data. The correlations between expression levels of hsa_circ_0014130 and various clinicopathological parameters of NSCLC were further analyzed by the Student *t* test or one-way analysis of variance (ANOVA). All tests were 2-sided, and *P* < 0.05 was considered statistically significant.

### Data availability

The datasets generated during and/or analysed during the current study are available from the corresponding author on reasonable request.

## Electronic supplementary material

supplementary information

Supplementary Dataset
