# Supplementary material for: Microarray profile of circular RNAs identifies hsa_circ_0014130 as a new circular RNA biomarker in non-small cell lung cancer
Source: Sci Rep. 2018 Feb 13;8:2878. doi: 10.1038/s41598-018-21300-5 (PMC5811528; doi:10.1038/s41598-018-21300-5)
Supplement: Supplementary file 1 — supplementary information [file 41598_2018_21300_MOESM1_ESM.pdf]

A Title Page

Microarray profile of circular RNAs identifies hsa_circ_0014130 as a new circular RNA biomarker in non-small cell lung cancer

Shaoyan Zhang[1]*, Xiaoli Zeng[2]*#, Ting Ding[3], Lin Guo[1], Yulong Li[2], Songlei Ou[1] & Hui Yuan[2]

[1]Department of Thoracic Surgery, Beijing Anzhen Hospital, Capital Medical University, Beijing 100029, China.

[2]Department of Clinical Laboratory, Beijing Anzhen Hospital, Capital Medical University, Beijing 100029, China.

[3]The Sixth Clinical College, Capital Medical University, Beijing 100029, China.

# Correspondence and requests for materials should be addressed to Xiaoli Zeng. Department of Clinical Laboratory, Beijing Anzhen Hospital, Capital Medical University, Anzhen Road No. 2, Chaoyang District, Beijing 100029, China

Tel: +86 10 64456319; Email: greatzxl@163.com

*These authors contributed equally to this work.

Supplementary figure

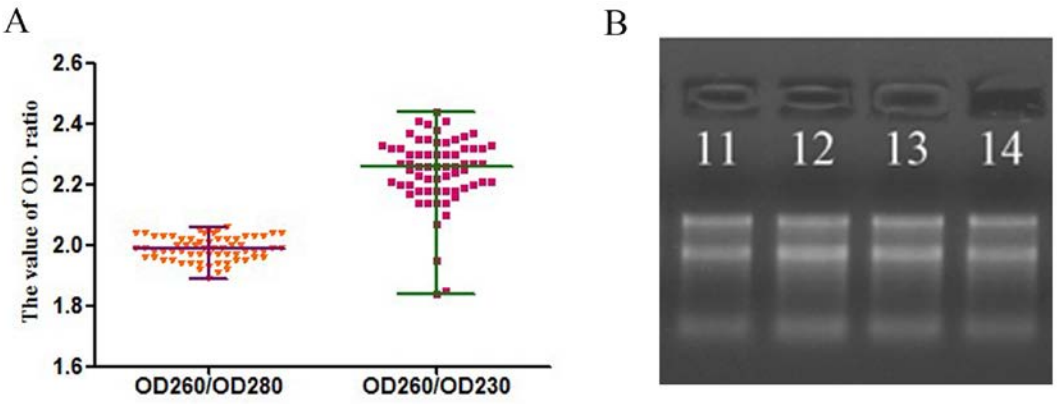

Figure 1. Validation of lung samples' RNA quality. (A) OD260/OD280 ratios should be between 1.8 and 2.1, OD260/OD230 ratios should be more than 1.8. (B) RNA Integrity and DNA contamination test by denaturing agarose gel electrophoresis. Sample 11 and sample 12 for NSCLC tissues; Sample 13 and sample 14 for adjacent non-cancerous tissues.

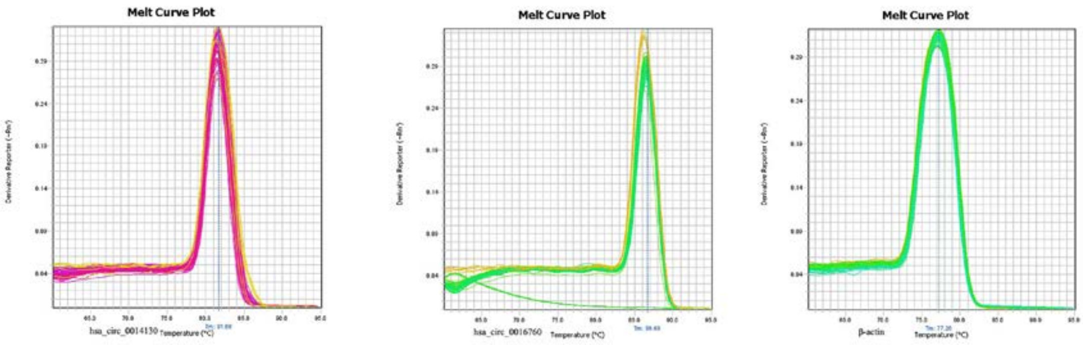

Figure 2. Melt curve plot of hsa_circ_0014130 and hsa_circ_0016760 and β-actin by performing RT-qPCR. The qPCR-amplified product yielded a single peak, indicating the specific amplifications.

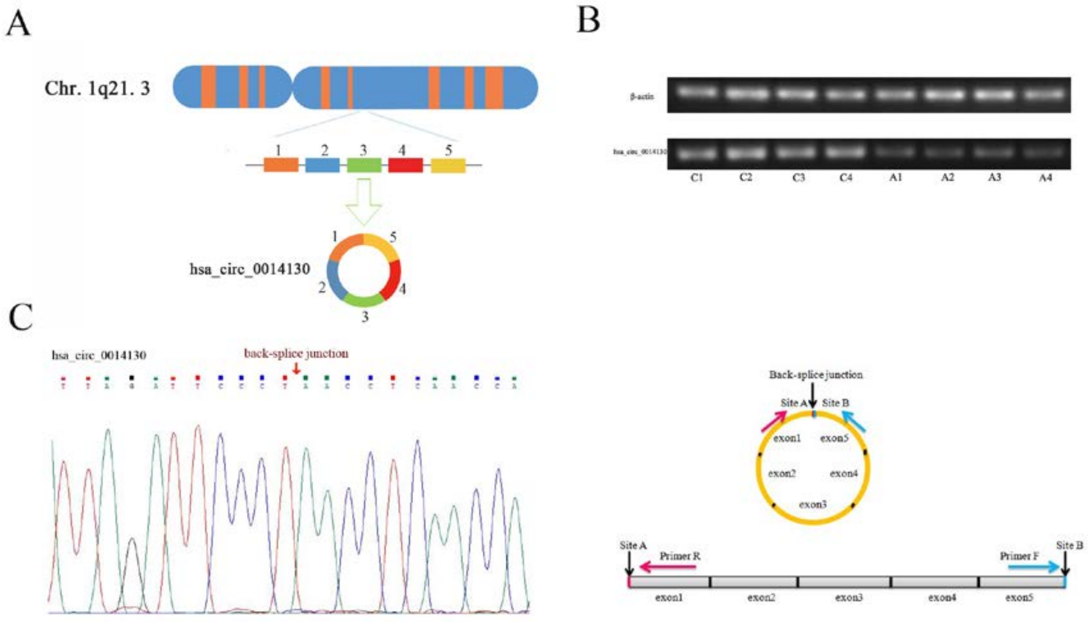

Figure 3. Characterization of hsa_circ_0014130 in NSCLC tissues and adjacent lung tissues . (A) Five exons form hsa_circ_0014130 by back splicing from chromosomal region 1q21.3. (B) qPCR products of hsa_circ_0014130 were subjected to gel electrophoresis. C1-C4, NSCLC tissues; A1-A4, adjacent non-cancerous tissues. (C) Sanger sequencing of hsa_circ_0014130 showed the back-splice junction (↓). Arrows represent divergent primers that bind to the genomic region of hsa_circ_0014130.

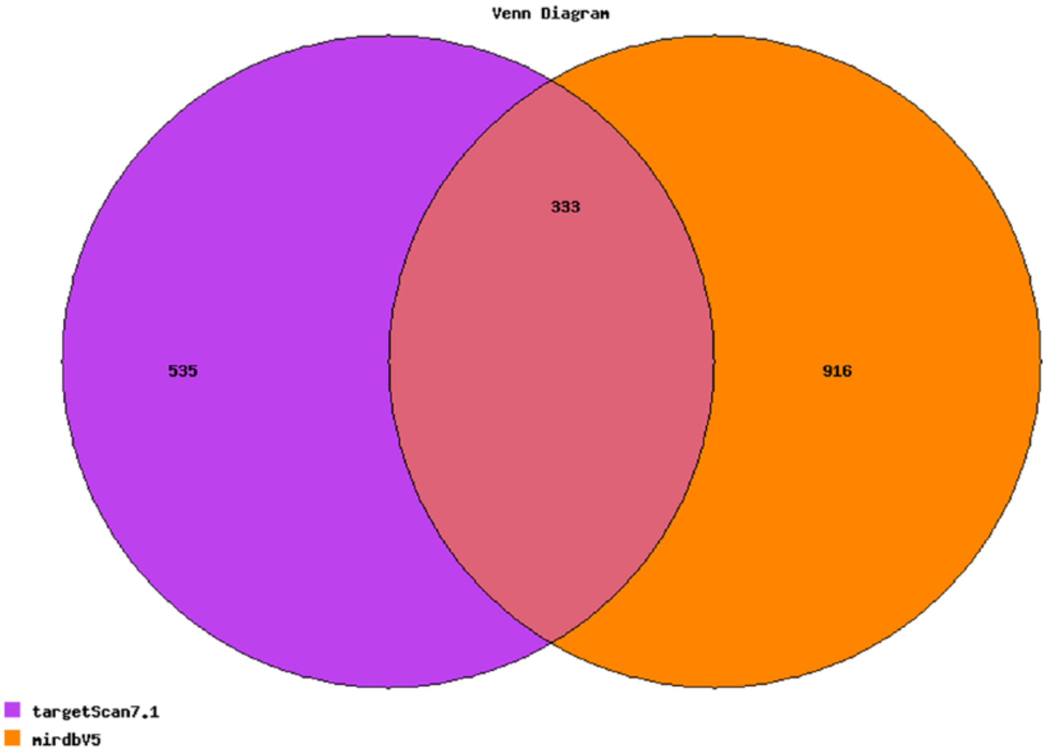

Figure 4. The predicted target genes of hsa_circ_0014130 based on circRNA-miRNA-mRNA gene co-expression network. Venn plot for predicting the number of mRNA target genes based on targetscan7.1 and mirdbV5.

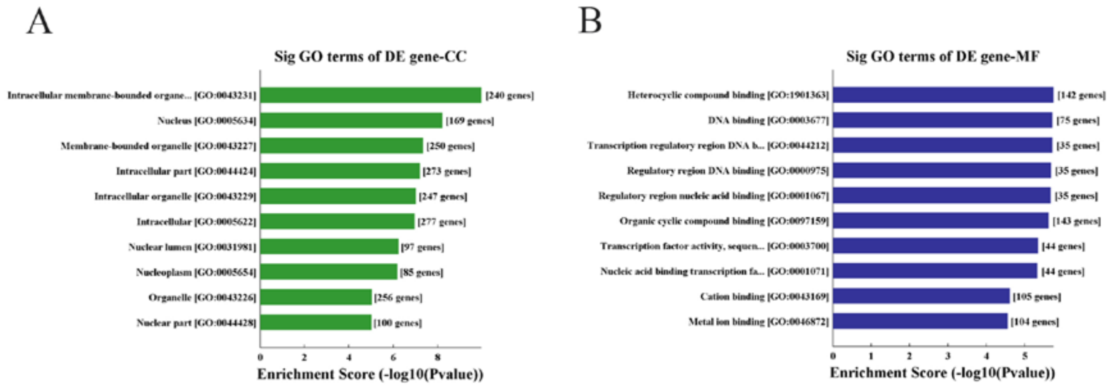

Figure 5. Gene ontology (GO) enrichment analysis for hsa_circ_0014130. The top 10 significantly enriched target genes and their scores (negative logarithm of P value) are listed as the x-axis and the y-axis, respectively. The horizontal axis represents the significant level of GOs. (A) GO enrichment analysis in terms of cellular components (CC). (B) GO enrichment analysis in terms of molecular functions (MF).

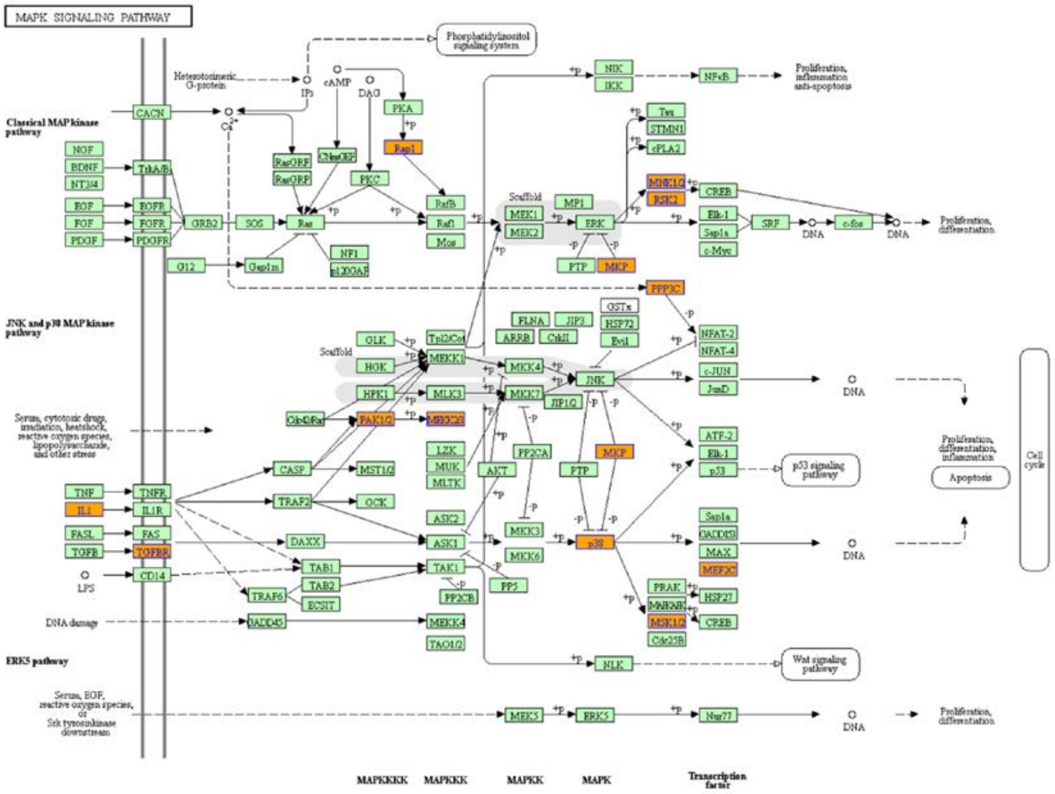

Figure 6. MAPK signaling pathway analysis for hsa_circ_0014130*. Orange marked nodes were associated with up-regulated genes, green nodes had no significance on MAPK signaling pathway. *This image was obtained from KEGG (http://www.kegg.jp/kegg/kegg1.html).
